# Teaching Near and Far – Broome, Western Australia

**DOI:** 10.7759/cureus.870

**Published:** 2016-11-09

**Authors:** Christina Herceg, Tia Renouf

**Affiliations:** 1 Aboriginal Medical Service, Broome Regional; 2 Emergency Medicine, Memorial University of Newfoundland

**Keywords:** rural medicine, australia, aboriginal, remote care

## Abstract

Broome is a remote coastal town in Western Australia. As a general practitioner working in Broome, I have been involved in the education of general practice trainee registrars both locally and remotely, as a supervisor with two different training programs.

## Editorial

Broome is a coastal town in the remote Kimberley region of Western Australia. It has a population of around 15,000, making it the largest town in the region. About a quarter of the population is Aboriginal [1]. Broome was developed around the pearling industry, and more recently, tourism and outlying mining industries have been important for the town. It is a beautiful and interesting, if sometimes challenging, place to live and work.

I work at the Aboriginal Medical Service in Broome. I have the privilege of teaching general practice (GP) registrars under the Australian General Practice Training Program (AGPT), resident medical officers, and medical students in this practice. I also work as a supervisor for the Remote Vocational Training Scheme (RVTS). RVTS is an Australia-wide organization that provides GP training for doctors in rural and remote areas, allowing them to stay in one community for the duration of their training. Other training programs, including AGPT, require that registrars change locations/practices at least once during their training. RVTS has recently expanded to include a stream for registrars in Aboriginal Medical Services.

These two supervisory roles are quite different. The AGPT registrars in my workplace come for six- or 12-month placements. They are employed as part of a regional Aboriginal health training program administered by the Kimberley Aboriginal Medical Services (KAMS) organization in partnership with the Western Australian General Practice Education and Training organization (WAGPET). More information about this program can be found in a recent review document [2].

Most registrars in our workplace are Australian-trained doctors. The GP registrars are supervised on-site by me or the other senior doctors, several of whom are also accredited supervisors. The registrars receive a considerable amount of face-to-face teaching, both formal and semi-formal tutorials, and “corridor teaching.” I am currently developing a series of tutorial topics to make our teaching more consistent and to ensure that common and important topics for our area and our patients, as well as for general practice more widely, are adequately covered. Both of the general practice colleges in Australia, the Royal Australian College of General Practice (RACGP) and the Australian College of Rural and Remote Medicine (ACRRM), have curricula that cover hundreds of pages; it is impossible to cover all areas of the curriculum during a registrar’s placement.

My role with RVTS is quite different. Most registrars in this program are international medical graduates (IMGs). They have widely differing previous experience and differing learning needs. RVTS provides weekly online tutorials, twice-yearly intensive face-to-face workshops, and medical educators who oversee their training. I have been a supervisor for trainees in Derby (220 km from Broome), Halls Creek (700 km away), and currently Kalgoorlie (2000 km away). Clearly, on-site supervision is not possible, though clinical teaching visits are arranged. There are arrangements for local support from senior doctors. I talk to my registrar via phone or Skype, initially once a week, then less often as their training progresses. These discussions may be about cases, specific topics, or general pastoral care. I see this as more of a mentor role, in contrast to the on-site daily supervision of AGPT registrars in my workplace. More about RVTS training programs can be found at www.rvts.org.au [3].

Technology has made this form of remote supervision and program delivery possible and has also made on-site teaching in general practice quite different than when I did my GP training. The use of online resources, online meeting/tutorial platforms, Skype, and even email communications have revolutionized training methods for registrars and also provide increased training opportunities for supervisors. Teleconferences, webinars, and face-to-face teaching are used for supervisor training by both WAGPET and RVTS.

Increased knowledge and research about clinical teaching and learning - the whole field of medical education - is the other area that has changed a great deal since I was a trainee and since I started teaching. There are many postgraduate courses on clinical education – I have completed a Diploma in Clinical Education at Flinders University in Adelaide, mostly through online learning, with some face-to-face intensive workshops. This is another example of relatively recent technology making training opportunities possible for those of us in rural and remote areas. Ever-increasing knowledge about how adults learn, differences in preferred learning styles, how doctors and other health professionals develop clinical reasoning skills, as well as research into curricula and assessments (“assessment guides learning”), have informed our efforts to improve on-site teaching and remote supervision.
